# The international classification of functioning, disability and health in clinical practice, research findings and their impact on training and education

**DOI:** 10.3389/fresc.2024.1420498

**Published:** 2024-09-12

**Authors:** Liane Simon, Friederike Gölz, Olaf Schenk, Thorsten Bührmann, Mathias Kauff, Olaf Kraus de Camargo, Stefanus Snyman, George Lüers, Britta Wulfhorst

**Affiliations:** ^1^ ICF Research Institute, MSH Medical School Hamburg, Hamburg, Germany; ^2^ Faculty Art, Health and Social Science, MSH Medical School Hamburg, Hamburg, Germany; ^3^ Faculty Life Sciences, MSH Medical School Hamburg, Hamburg, Germany; ^4^ Faculty of Human Science, MSH Medical School Hamburg, Hamburg, Germany; ^5^ CanChild Centre for Childhood Disability Research, Department of Pediatrics, McMaster University, Hamilton, ON, Canada; ^6^ Centre for Community Technologies, Nelson Mandela University, Gqeberha, South Africa; ^7^ Faculty of Medicine, MSH Medical School Hamburg, Hamburg, Germany

**Keywords:** ICF, interprofessional collaboration, teaching, training and education, implementation, research-development-innovation, health professionals, interprofessional education (IPE)

## Abstract

At the ICF Research Institute (at MSH Medical School Hamburg) multiprofessional experts collaborate on various research projects with a focus on bio-psycho-social health and education. Initially, the main goal was monitoring and evaluating the implementation of the International Classification of Functioning, Disability and Health (ICF) in clinical practice. Over time and based on the initial findings, the research group started developing new approaches to support training and education of health professionals in the use of the ICF. As a result, substantial changes have recently been made in the curriculum and structure of several courses to improve and expand interprofessional teaching at the MSH Medical School Hamburg (MSH). Furthermore, creative didactic approaches in combination with interprofessional education have been developed.

## 1 Introduction

Describing and classifying health and health-related states is a key challenge for health-professionals as well as researchers and policy makers. The International Classification of Functioning, Disability and Health (ICF) provides a comprehensive and internationally recognized framework that incorporates not just physical aspects of health but also social and environmental factors (1).

In this article, we will present an overview of research conducted by the ICF Research Institute (IRIs) at MSH Medical School Hamburg (MSH). Building on the results of these projects we will discuss the importance of interprofessional education for the implementation of the ICF and portray examples of measures that support interprofessional competencies among health students.

Research at IRIs is dedicated to the study of the bio-psycho-social model of functioning, disability, and health (1). Professionals from various departments at MSH collaborate in interdisciplinary research groups to gain a better understanding of bio-psycho-social aspects of health and education. Health is not only viewed at an individual level but over a comprehensive range of bio-psycho-social and environmental factors that impact individuals’ and societal well-being. Consequently, IRIs researchers explore strengths- and community-based approaches to promote and enhance health and well-being. They also aim at translating and implementing research findings into clinical practice. One goal is to foster interdisciplinarity and interprofessionalism in the fields of health, education, and social services. In this article, we will present findings from three distinct research projects that have the potential to be beneficial in this regard.

Moreover, IRIs’ goal is to foster interprofessional competencies among future health professionals. Building on the findings of the aforementioned research projects we will present an overview of our approaches to promote interdisciplinarity and interprofessionalism among students—based on the bio-psycho-social model of the ICF.

## 2 ICF in clinical practice

The following findings result from three different research projects conducted by members of IRIs: “ICF Mapping”, “ICF in Nursing” and “I-Teams”.

### 2.1 Project 1: ICF Mapping

ICF Mapping is a research project that has been conducted in various phases since 2016. Initially, the primary emphasis was on monitoring the implementation of the ICF in early childhood intervention centers (EIC) in Germany, with the aim of assessing the extent of ICF usage across all EICs in Germany. The pilot study in 2016 was exploratory in design and included both open and closed questions to assess the implementation status of the ICF in early childhood intervention centers. A paper-and-pencil questionnaire was distributed to staff members at 14 EICs in Hamburg and resulted in 49 completed questionnaires. The responses were analyzed qualitatively using content analysis, and categories were formed inductively (2). Based on the findings of the pilot study, we developed a questionnaire with seven closed questions. We sent a link of the online survey to all leaders of EICs in Germany (*N* = 1000). Data collection took place in the summer of 2017. 329 questionnaires were returned, rendering a response rate of 32.9%. The results were presented at the WHO-Family of International Classifications annual Meeting 2017 (3). The online surveys were then repeated annually until 2020.

The project then proceeded to evaluate the learning and training needs of professionals with the aim to identify the barriers and facilitators that influence ICF implementation. Since the enactment of the Federal Participation Act (Bundesteilhabegesetz—BTHG) in Germany, which made ICF usage mandatory for rehabilitation and social institutes starting in 2018 (4), the adoption of ICF in EICs has become more widespread. Initially, there were only a few ICF-based methods and instruments available. However, over time, several ICF-based needs assessment tools have been developed, and the ICF Mapping project has focused on their design and application (5).

Throughout the various phases and in light of the findings, a new question emerged: Do we need to cultivate a new kind of interprofessional culture to effectively communicate in this “common language” called ICF? Subsequent phases aimed to address this.

**Figure 1:**
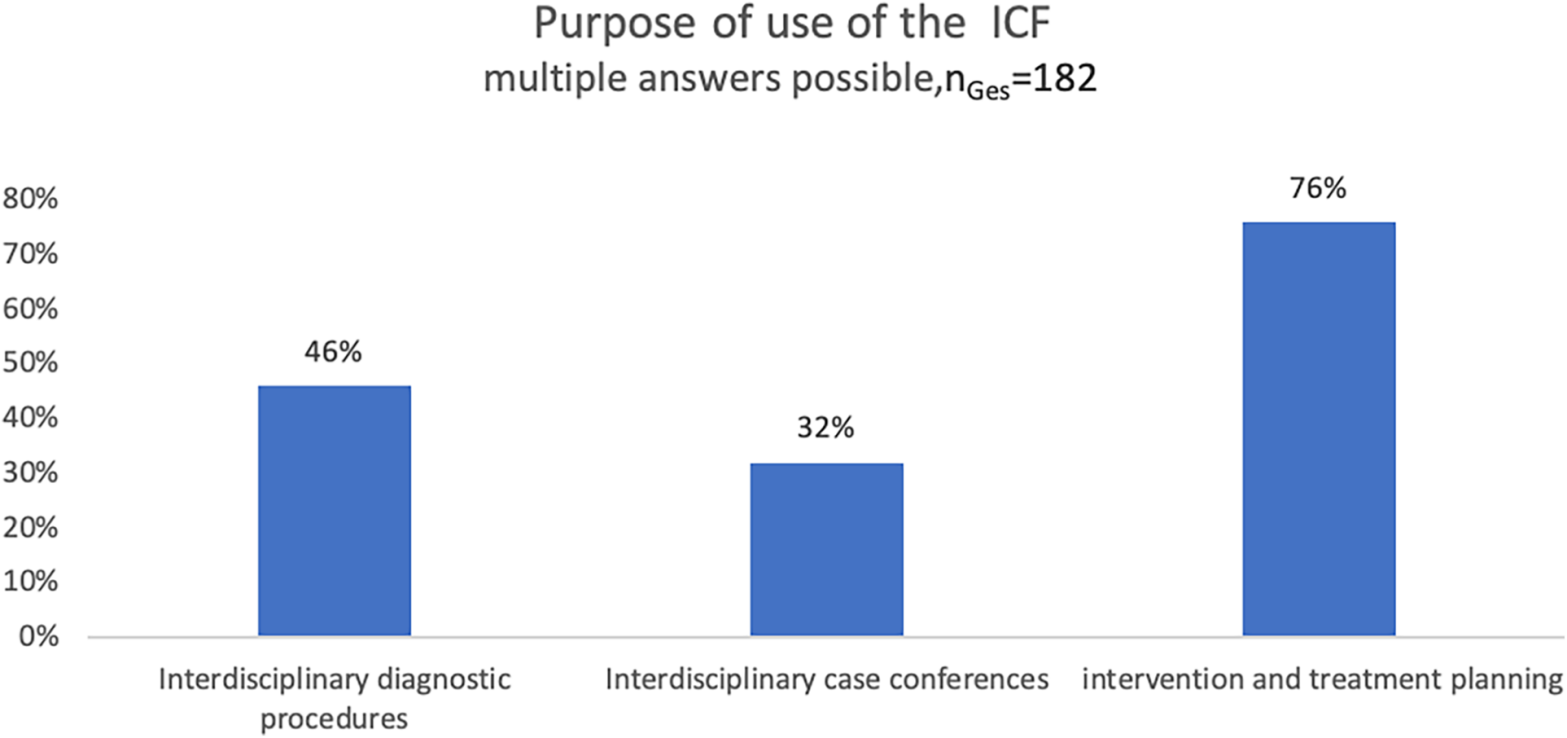
Purpose of the use of the ICF in EICs in Germany, online survey 2020.

**Figure 2:**
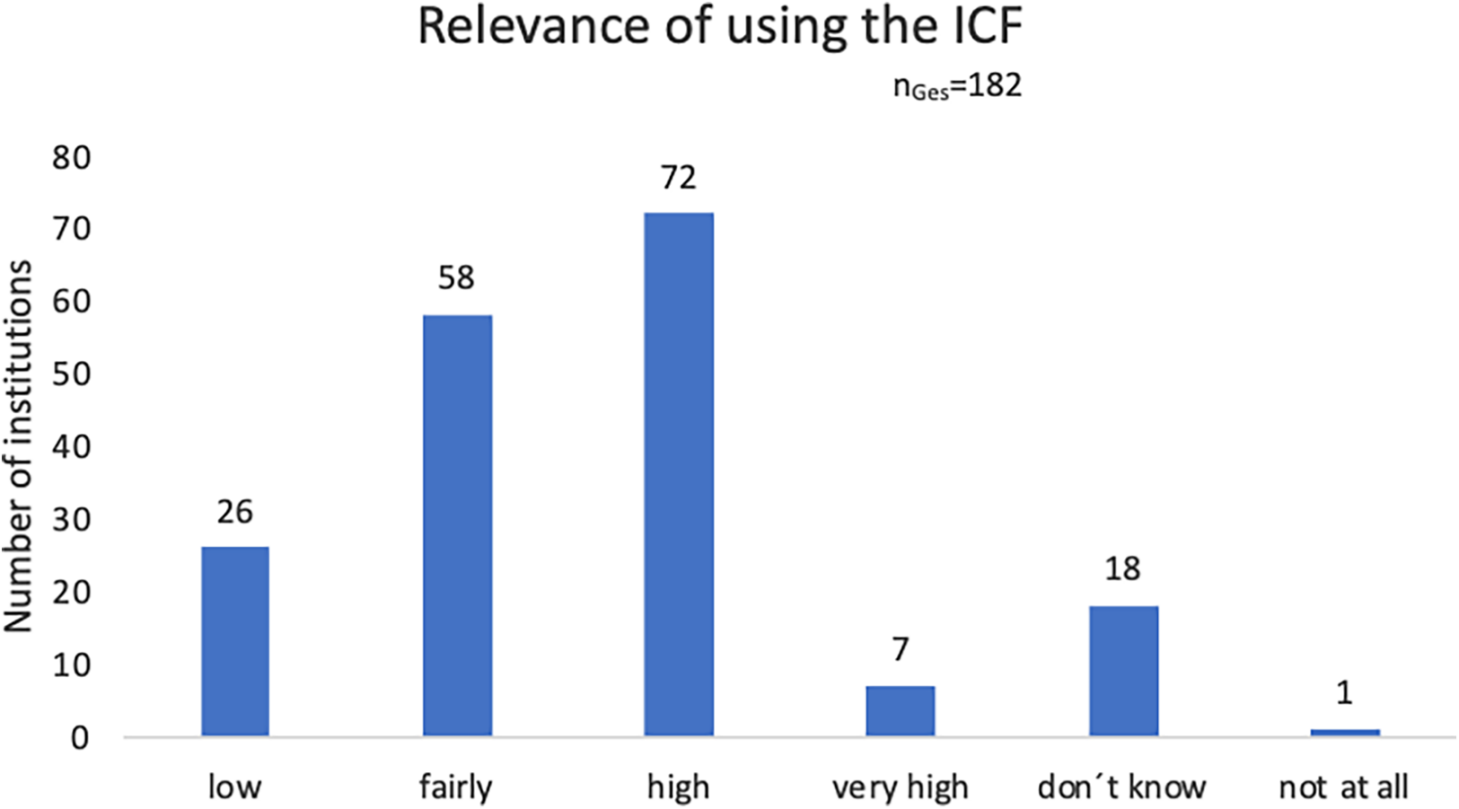
Estimated relevance of using the ICF, online survey 2020.

**Figure 3:**
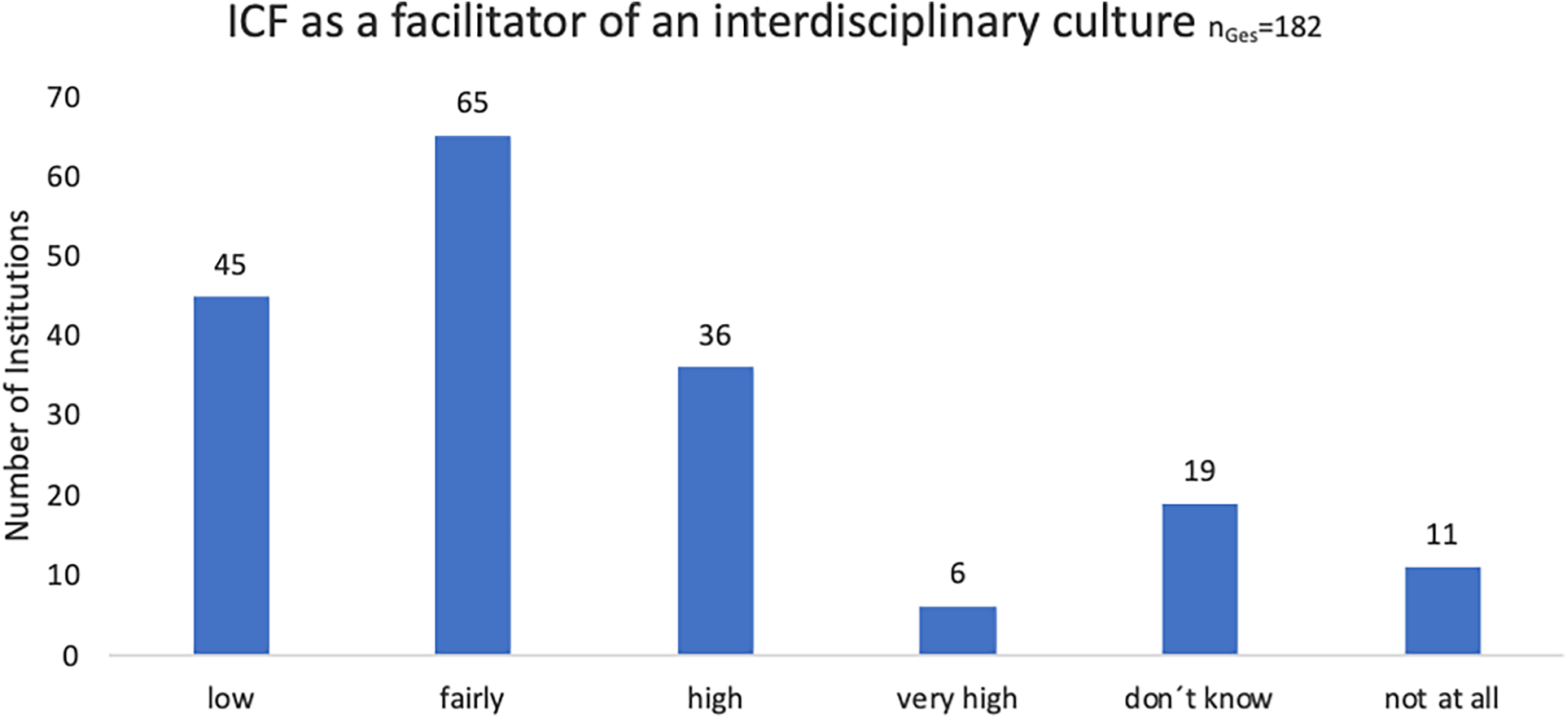
Perceived support of an interprofessional culture by using the ICF, online survey 2020.

In 2020, the above-mentioned online survey was expanded by additional questions. A total of 182 questionnaires were returned. The responses to questions concerning the setting and relevance (“What do you use the ICF for” and “I assess the relevance of using the ICF as”) were as follows (Figures 1, 2):

Additionally, two questions were included to assess the perceived support of an interprofessional culture through the utilization of the ICF (“Would you say that using the ICF fosters an interdisciplinary culture?) (Figure 3).

Responses to an additional open question in the survey, which explored how the use of the ICF facilitates an interprofessional culture, were analyzed using a qualitative approach (6). The answers (*n* = 146) were categorized as follows:

25% Interprofessional communication (e.g., “facilitates increased communication across professions”)
21% Common language (e.g., “we share a common language”)
15% Participation-oriented (e.g., “joint focus on participation”)
14% Other (e.g., “not enough experiences yet”)
13% Not at all (e.g., “Supposedly standardized language, but I don't always experience it that way”)
12% Needs assessment (e.g., “joint use of needs assessment tools”)

These categories provide valuable insights into the ways in which the use of the ICF supports the development of an interprofessional culture.

Subsequently, these results have been discussed by experts in two different focus groups (2022). The focus groups consisted each of 5 professionals working in EICs in various federal states in Germany. Both groups also included individuals who were not yet familiar with the ICF, but primarily those who already had experience in its application.

Their discussions of the preliminary results were transcribed and analyzed using summarized content analysis techniques (6). The findings were as follows (7):

▪ Although the use of the ICF can promote interdisciplinary collaboration, we recognized that there is initially a barrier that must be overcome. This is because a common language must first be learned. Initially, no one knows why they should bother with the ICF.
▪ Once this hurdle is overcome, it can make life easier, especially regarding participatory assessment, discussion, goal and support planning as well as interprofessional collaboration.
▪ It becomes rewarding to use the ICF when a different mindset is established, emphasizing participation-oriented interdisciplinary collaboration.

The project taught us that although there is value in using the ICF in different interdisciplinary settings and the application is felt as helpful to determine intervention goals collaboratively, it still only promotes an interdisciplinary culture to a certain extent.

### 2.2 Project 2: ICF in Nursing

This project is being carried out in collaboration with Ward 11 of the Child and Adolescent Psychiatry Department at the Evangelical Hospital Alsterdorf in Hamburg since 2023. Here, preschool children (6 months—6 years) are admitted for treatment together with a parent. In addition to medical and therapeutic care, parents and children receive intensive support from nursing professionals through a concept called “primary nursing care”. As part of this project, primary care nurses were introduced in ICF and started interviewing parents at the beginning of their child's hospitalization about their expectations for treatment, what is going well, and what is not going well. These questions aimed to implement the person-centered approach of the ICF while also fostering interdisciplinary exchange (8). The information obtained from the nursing professionals in this manner was incorporated into the handover discussions with the treating therapists, psychologists, and physicians, which occur daily in this unit.

The implementation of the ICF as well as the method of parent interviews in the first days of inpatient admission is scientifically accompanied in various steps, by a mixed methods approach to answer the research question: “Does the implementation and use of the ICF (in the form of participation-oriented questions that the primary care nurses ask the parents during the first week of their inpatient stay in the child and adolescent psychiatry) change the satisfaction and professional attitude of the nurses?”

At the level of the direct target group, a paper-and-pencil questionnaire administered to the primary care nurses (*n* = 13) at different measurement points is supplemented by semi-structured interviews with a subset of the nurses (*n* = 5) to explore individual conditions affecting their satisfaction and professional attitude.

Participants report their experiences, challenges, and perceived changes in professional practice since the implementation of these parent interviews. As this project is still ongoing, we can only show some preliminary results:

▪ By taking on new tasks related to person-centered parent conversations based on the ICF, primary care nurses develop a more professional attitude and a deeper understanding of their role.

*“Because then you have a better overview for yourself and the goal becomes clearer for oneself as well: ,What can we work on? “Often, this also leads to: ,What can we do there, how can we help you?””*.

▪ The person-centered approach promotes improved communication between primary caregivers and parents, leading to higher satisfaction and a sense of appreciation among parents.

*“I think that saves us a lot of time, because sometimes we talk past each other for two weeks until we finally meet and know, “Ah, so that´s what it´s really about”. Exactly that´s why I find it very valuable”*.

▪ Furthermore, the integration of ICF principles leads to better collaboration among different professions and more efficient care.

*“This lays a cornerstone, through which we all then come into conversation more quickly”*.

▪ Nursing professionals also reported a positive change in their perspective, moving towards a more holistic view of individuals and families, and away from stereotyping. This new perspective was described as another positive outcome of integrating ICF into their professional practice.

*“Working with these ICF based questions has really changed my perspective. One becomes somehow sensitized to such resource-oriented work and broadens onés perspective by not only looking at it like: This is a ,behavioral child‘ or this is an ,eating child‘ and we have the problem X and we have to make sure that the problem is solved, but to look at the entirety: What has happened in the past? How are they socially positioned? What network do they have and what biographic baggage do they carry?”*

The positive effects of integrating ICF-based parent interviews in the professional practice of nursing professionals underscore the importance of a holistic and resource-oriented approach to patients and families. These first results suggest that training in ICF-based parent interviews not only influences direct nursing practice but also enhances the confidence and professionalism of nursing professionals. Respondents reported feeling more confident in managing complex situations and better able to recognize and respond to individual needs. The introduction of ICF-based parent interviews was found to increase parental involvement in the therapy process, as they could contribute more to decision-making and felt better understood.

From this project we are learning that the implementation of the ICF into an inpatient setting has a positive impact on both healthcare professionals and families. We conclude that a greater dissemination of the ICF in different healthcare sectors would be beneficial to the workforce and patients.

### 2.3 Project 3: I-Teams

In this project, a new concept of inclusive early intervention was trialed and evaluated (2020–2023).

One of the main parts of the new concept was to install interdisciplinary case discussions (I-Teams). Those I-Teams were initiated for each child in the Herzberg Early Intervention Center. Invited were all professionals (e.g., early interventionists, educators, physicians, physio-, occupational-, speech therapists) involved in the child's care or treatment. The I-Teams were not only interdisciplinary but also interinstitutional because professionals from several institutions came together before treatment started to discuss needs and goals. What was new about it was that:

1. Every professional—not only those who were employees of the Herzberg center—had the opportunity to bill for these joint discussion times.
2. Parents were equally involved in the discussions as advocates for their children.
3. The discussions were structured in accordance with the ICF.

Data collection and analysis were conducted using a mixed methods approach, both qualitatively and quantitatively. In addition to the quantitative collection and analysis of key figures before, during, and after the implementation of the new concept, workshops, group discussions and interviews with professionals were conducted. The parents participating in the I-Teams were contacted and asked to complete paper-and-pencil questionnaires, which included scoring methods, regarding their experiences and evaluations.

Initial results show that both professionals and parents consider these joint discussions as beneficial and view them as valuable:

▪ “*So, it (the collaboration) has become more structured, and I would also say it has become more intensive.”*
▪ *“Everyone can express their point of view.”*

Especially parents (*n* = 16) rate the I-Teams very positively. The following statements were endorsed by parents:

▪ The professionals engaged in the “I-Team” for collaborative and open communication with each other and with us“ (rated as “strongly applies” by 12 persons out of 16)
▪ The professionals worked with us to align the goals and plan (rated as “strongly applies” by 10 persons out of 16)
▪ We felt involved and taken seriously (rated as “strongly applied” by 14 persons out of 16).

This study expanded our understanding of the use of the ICF beyond only one institution serving as a unifying framework across sectors.

### 2.4 Research conclusions

Overall, these initial findings suggest that the use of the ICF promotes both interdisciplinary collaboration and participatory decision-making processes. This confirms the objectives set forth by members of the WHO-Family of International Classifications during the development of the ICF in 2017, that is to establish a common language and promote a person-centered approach (1, 9).

However, the results also highlight the challenges associated with implementing the ICF for professionals and organizations alike. Anchoring the ICF as a fundamental tool often entails organizational change processes, presenting significant hurdles. This includes establishing a professional culture of interdisciplinary collaboration. Interdisciplinary collaboration among health professionals often relies on individual efforts and is thus left largely to chance (10). There are few systematic, institutionalized forms of interprofessional and interinstitutional collaboration. Professionals within each discipline often use a professional language specific to their field, which can hinder communication with counterparts from other disciplines (11). The ICF can serve as a bridge here, but it requires learning this common language (8).

One opportunity to learn this common language lies in the interprofessional education of health professionals. Well-functioning interprofessional cooperation requires not only interprofessional competencies among team members but also a common understanding and identification. We believe that joint study courses between students from different health-related disciplines enables intergroup contact and can foster joint identification as health professionals and reduce mutual prejudice and negative stereotyping between disciplines (12). Moreover, experiencing successful cooperation might facilitate beliefs in the value of (interprofessional) diversity (12). Students could gain initial interprofessional experiences, learn the value of exchange, and acquire the common language during their training. To fundamentally promote these developments on a larger scale, IRIS research aims to foster interdisciplinary and interprofessional collaboration within (and beyond) MSH based on the biopsychosocial model of the ICF (1), as elaborated in the following chapter.

## 3 Interprofessionalism in the education and training of health professionals—From study programs to third mission

Universities have expanded their objectives beyond teaching and performing research but are also expected to contribute to society in general, their so-called third mission (13). At MSH we develop, implement, and evaluate curricular and extracurricular cross-faculty and cross-degree teaching/learning formats for students, as well as workshops for staff, preferably with the involvement of international partners. Through this collaboration and exchange with experienced practitioners, teaching and research continuously evolve to optimize current practice in healthcare. This process, which integrates research into practice, can be referred to as a “Learning Health Education System”.

### 3.1 Competency oriented education

A paradigmatic orientation based on the fundamental premises of the ICF and the CanMEDS roles model was adopted for the following outlined examples in the academic education of health professionals (see Chapters 3.3.1–3.3.4). The primary focus is on the fundamental orientation towards the bio-psycho-social concept of health as adopted by the WHO, which also underpins the ICF. The ICF classification is of great relevance to all health professions: “The overall aim of the ICF classification is to provide a unified and standard language and framework for the description of health and health-related states. It defines components of health and some health-related components of well-being (such as education and labor). The domains contained in ICF can, therefore, be seen as *health domains* and *health-related domains*” (1, p.3).

Further operationalization can be achieved by referencing framework models for competency development in health professions. The CanMeds roles model appears suitable for this purpose (14). Sottas (15) has adapted the concept to all health professions. As a result of the CanMEDS project to further develop the competency framework, Frank et al. (2015) highlight the successful transfer of the concept to other health professions: “CanMEDS is now used in dozens of countries on five continents, in medicine and in other health care professions, making it the most recognized and most widely applied health care profession competency framework in the world” (14, p.5).

This is also aligned with the Lancet Report “Education of Health Professionals for the 21st Century” from 2010 (16). Competency-driven approaches need to be incorporated into education, and interprofessional and cross-professional learning should be promoted to overcome unnecessary hierarchies and foster collaboration in teams. With regard to the application level of interprofessional collaborative competencies to be acquired in educational processes, WHO defined interprofessional collaborative practice (IPCP) in 2010 as follows: “IPCP in health-care occurs when multiple health workers from different professional backgrounds provide comprehensive services by working with patients, their families, carers and communities to deliver the highest quality of care across settings” (9, p.13).

Interprofessional education (IPE) is needed to apply interprofessional competencies in practice: “Occasions when members or students of two or more professions learn about, with and from each other, to improve collaboration, and the quality of care and services” (17). IPE, in turn, can only lead to the core outcome if Competencies for Interprofessional Collaborative Practice (CIPCP) are instilled as part of the educational processes: “The integrated enactment of knowledge, skills, values, and attitudes that enable working together successfully across the professions and with patients, along with families and communities, to improve health outcomes in specific care contexts” (18, p.8). The use of the ICF in the education of health professionals ensures that all professions have a common foundation on which to build. This promotes better understanding and closer collaboration already during training. To develop interprofessional competencies in practice among health professionals whose training did not include such concepts, two roles from the above mentioned CanMeds model (14) seem particularly central. Foremost is the role of the “Scholar”. Additionally, the role of the “Collaborator,” in terms of implementing interdisciplinary and interprofessional higher education didactic concepts, is relevant.

### 3.2 Didactic concepts for the training of health professionals

The above-mentioned Lancet Report clearly identified the need to implement transformation processes in health professions education. In particular, it highlighted that training objectives and content were not aligned with societal needs, that there was a lack of teamwork, that there was hierarchization by profession, specialization and gender, that there was a technical-instrumental approach without an understanding of larger contexts and systems of care, that there was a quantitative and qualitative imbalance between the supply and demand for health professionals, and that, among other things, incomplete, outdated and rigid curricula resulted in inadequately prepared graduates being released into the health care system (16). In their recent update (19) the authors analyze transformative developments since 2010, which they say have included competency orientation, the initiation of interprofessional competencies, and the use of information technology in health professions education.

Competency-based education of health professionals was the subject of 24% (105 of 437) of the studies in the review conducted by Frenk et al. (19). These addressed the use and improvement of competency-based education. The concept of competency encompasses a broad range of skills that combines complex cognitive abilities with specific skills (20). With respect to the initiation of competencies for interprofessional collaborative practice, the studies considered a wide range of topics relevant for the implementation of IPE (19). Particularly relevant to the focus of this paper are Frenk et al.'s (19) comments on studies that address the initiation of critical thinking skills, training, and identity. For example, these are studies that consider leadership skills critical to success (21–23) and studies that focus on uniprofessional vs. interprofessional identity (i.e., identity as a health care professional vs. identity as a team) (24, 25).

### 3.3 Examples to promote interprofessional competencies

The following presents examples of measures implemented within the teaching framework at MSH Medical School Hamburg to promote interprofessional competencies within the framework of curricular offerings, as well as offerings within the context of continuing education for currently active health professionals. The paradigmatic fundamental orientation for the didactic implementation of these measures is—as outlined above—the WHO's adoption of the bio-psycho-social model and the ICF (1) derived from it.

#### 3.3.1 Example 1: Problem Based Learning interprofessional Day (PBL-i-Day)

Problem Based Learning is often highlighted as a suitable method for initiating interprofessional competencies (26). Therefore, once per semester, the university becomes an interprofessional learning landscape. Students from all programs that train health professionals in a broad sense (in addition to academically trained health professions i.e., therapeutic professions, nursing, psychology, and human medicine, degree programs with strong connections to health promotion, prevention, diagnostics, therapy, rehabilitation, and management are also considered part of the group of Health Professionals. This includes degree programs in healthcare controlling and management, social work, early childhood intervention, sports sciences, and artistic-therapeutic studies.) work in mixed (interprofessional) groups on PBL (Problem-Based Learning interprofessional – PBL-i) cases that address issues in future healthcare provision. For instance, the consequences of AI or problems in cross-sectoral care are discussed. The PBL-i groups are supported by certified student tutors who also moderate the presentation of results in the plenary session. The design of the PBL-i cases, which is also done by students within the framework of a course, as well as the tutorial support of the PBL-i groups and the moderation of plenary sessions, are intended to foster leadership competencies. The design of the cases, in turn, is aimed at promoting critical thinking skills, which are further challenged by the integration of different disciplinary approaches to the respective cases.

#### 3.3.2 Example 2: interdisciplinary mandatory and elective modules

Starting from interdisciplinary extracurricular elective module offerings that could be attended in addition to the regular study program, interdisciplinary elective modules have now been implemented in all curricula. This implementation is a central prerequisite for the participation of all students, as existing structural, organizational, and motivational barriers allowed only a small group of students to engage voluntarily. Moreover, modules that are anchored in several curricula (e.g., Ethics) are offered across different study programs. Students who take additional modules beyond the mandatory interdisciplinary modules and measures (e.g., PBL-i Day) or participate in extracurricular interdisciplinary measures (e.g., interdisciplinary lecture series) can, upon reaching a minimum number of hours and submitting a portfolio, obtain a certificate “Interprofessional Collaboration Expert in Health Care”.

#### 3.3.3 Example 3: interdisciplinary and interprofessional competency development in lecturers

A central aspect of promoting interdisciplinary and interprofessional competencies among students is the preceding competency development in lecturers. When students from various disciplines simply experience a module concept together in a seminar room without it being specifically designed for interprofessional requirements, the effects will remain minimal. For this reason, a tiered program for engaging with interprofessionalism and interdisciplinarity in teaching is offered to faculty members. From individual workshops to the option of enrolling in more comprehensive modules, a further educational university pedagogy master's program “Medical and Health Education” is also offered, which explicitly addresses an interdisciplinary approach to educational issues in the training of health professionals. The conception of the Master in Medical and Health Education program is based on three central structural principles that are longitudinally incorporated into the individual modules:

• Competency orientation (competency-based medical education—CBME)
• Interdisciplinarity and Interprofessionalism
• CanMEDs framework concept, especially the roles of “Scholar” and “Collaborator” (14) The professional medical roles described have been adapted by various authors to other health professions (27, 28) and specific areas of responsibility (Interprofessional Cooperation) and the concept has been extended to all health professions by Sottas (15). As a result of the CanMEDS project for the further development of the competency framework, the authors around Frank highlight the successful transfer of the concept to other health professions: “CanMEDS is now used in dozens of countries on five continents, in medicine and in other health care professions, making it the most recognized and most widely applied health care profession competency framework in the world” (14, p.5).

#### 3.3.4 Example 4: aesthetic practice as an approach for teaching interprofessional collaboration

A special focus is on the role of aesthetic practice as an approach within interprofessional education in order to acquire a deeper understanding of interdisciplinary collaboration. Our fast-paced era seems to be highly demanding to health professionals. They are increasingly confronted with uncertainties and the unpredictable. They require self-reflectiveness, creativity, flexibility, intuition, dialogic skills, responsiveness, openness to results, and the willingness for interprofessional collaboration (29).

To support future health professionals, it has been proposed to foster creativity, critical thinking, and social competence as prerequisites for independent and responsible action in their professions (30). According to Klafki (31), students should be empowered for self-determination, emancipation from external determination, as well as the ability for autonomy and freedom of thought. The idea of artistic-aesthetic engagement and the associated self-experience in health professions education in addition of providing and receiving feedback in aesthetic creation aim to facilitate the development of the required core competencies.

Based on this conviction, the Faculty of Art, Health and Social Science at MSH Medical School Hamburg has emphasized the importance of arts in the development of interprofessional competence. Aesthetic education and practice are established in all programs at this faculty, justified by the necessity to prepare students for practice, enabling them to react creatively and flexibly in seemingly intractable situations.

The main goal of adding aesthetic practice to interprofessional education is to create moments of knowledge and experience for collaboration in interprofessional teams. This approach was introduced at the 2022 Interprofessionalism—Interdisciplinarity conference at MSH Medical School Hamburg involving academic university employees from MSH and other universities.

Participants representing various professional backgrounds, engaged in collaborative art creation, followed by reflection on the process and its implications for interprofessional competence development as exemplified below:

Step 1: Participants were tasked with creating a coherent image together within a set time. Analogous to their professional everyday life, the task described a theme or problem that needed to be collectively negotiated and represented. As a constraint, participants were instructed not to communicate verbally with each other during the process but to interact aesthetically and artistically. Impulses of affirmation, contradiction and positioning were to be conveyed through actions and creative interventions. They were also advised to keep the entire image in mind and not show separate individual positions at the end of the process. The goal was to strive for an independent work, like a seamless piece with a unified handwriting, where individual positions connect to form a complete work or a comprehensive statement.

Step 2: The created artwork and its process were collectively examined, considering visual phenomena such as intersections, compressions and open spaces. Participants reflected on individual contributions and challenges.

Step 3: Participants reflected on their competency development in interprofessional collaboration noting the non-hierarchical, creative and process-oriented nature of the aesthetic practice, fostering a better understanding of different perspectives.

## 4 Conclusions

Findings from research projects at the IRIS Institute at MSH Medical School Hamburg show, that the use of the ICF supports the development of an interprofessional culture by providing a common language that helps health professionals to understand and talk to each other. Moreover, there were hints, that their focus shifted from a purely functional and biomedical view to a person-centered approach taking the persons opportunities to participate in life into account. Additionally, with the help of the ICF, both interdisciplinary collaboration and participatory decision-making processes are promoted.

Although the use of the ICF can promote interdisciplinary collaboration, we also recognize that there is initially a barrier that must be overcome. This is because to learn the common ICF language it is not enough to become familiar with the terminology, it also requires to become familiar with the culture that this new language represents. Brown stated: “A language is a part of a culture, and a culture is a part of a language; the two are intricately interwoven so that one cannot separate the two without losing the significance of either language or culture” (32, p. 165).

That's why IRIs Institute started to provide professional development and continuous reflection and further development of the interdisciplinary and interprofessional university concept over the years, which is now being gradually implemented and further developed. The dimensions considered include study programs, the continuing education and training of our faculty members, and, in the sense of the Third Mission, the continuing education and training of already practicing health professionals. The presented examples for implementation in the teaching framework at MSH Medical School Hamburg—POL-i-Day, interdisciplinary elective modules, inclusion of aesthetic education tools—are intended to prepare future health professionals for collaboration based on the bio-psycho-social model of the ICF.

Interest in interdisciplinary collaboration can be sparked through creative processes, so, from our perspective, artistic and creative approaches in combination with interprofessional education can be a valuable and meaningful addition to interdisciplinary university teaching for future health professionals.

The effectiveness of the concept outlined here is to be assessed. This makes the practice at the MSH itself the object of research, through which results can be generated as described in Section 2.

## Data Availability

The raw data supporting the conclusions of this article will be made available by the authors, without undue reservation.
